# Measuring Food Waste and Consumption by Children Using Photography

**DOI:** 10.3390/nu11102410

**Published:** 2019-10-09

**Authors:** Agnes Giboreau, Camille Schwartz, David Morizet, Herbert L. Meiselman

**Affiliations:** 1 Institute Paul Bocuse Research Center, 69130 Ecully, France; camille.schwartz@inra.fr; 2 Bonduelle Corporate Research, 59650 Villeneuve-d’Ascq France; david.morizet@rd.loreal.com; 3 Herb L. Meiselman Training and Consulting, Rockport, MA 01966, USA; herb@herbmeiselman.com

**Keywords:** food waste, school lunch, photography, vegetables, liking, French meals

## Abstract

A photography method was used to measure waste on food trays in school lunch in France, using the 5-point quarter-waste scale. While food waste has been studied extensively in US school lunches, the structure of the French lunch meal is quite different, with multiple courses, and vegetables (raw and cooked) in more than one course. Vegetables were the most wasted food category as usually seen in school lunch research, especially cooked vegetables, which were wasted at rates of 66%–83%. Raw vegetables were still wasted more than main dishes, starchy products, dairy, fruit, and desserts. Vegetables were also the most disliked food category, with the classes of vegetables falling in the same order as for waste. Waste and liking were highly correlated. Sensory characteristics of the food were cited as a main reason for liking/disliking. There is a strong connection between food liking and food consumption, and this connection should be the basis for future attempts to modify school lunch to improve consumption. The photographic method of measuring food waste at an individual level performed well.

## 1. Introduction

The measurement of food consumption and food waste are important for the goals of healthy eating and sustainability. Much of the research on measuring food intake and food waste has been directed to children [1]. One of the major challenges with children’s eating is the consumption of vegetables, which have received a large share of the attention in research [2,3,4,5].

The present study was designed to extend the research on school food waste to French school canteens using the photographic method of estimating waste and study the reasons for non-consumption with particular attention on the differences between the various foods served in the French meal pattern. Much of the research on food consumption and food waste comes from studies of school lunches in the USA, including served hot lunches, served cold lunches (sandwiches, etc.), packed lunches from home, and possibly packed lunches purchased in shops. The children in the USA do not typically eat a hot lunch when they are at home, where sandwiches prevail [6]. Sandwich lunches also tend to be the norm in some Nordic countries [7,8]. Further, the meal content of the French mid-day meal and of the school mid-day meal are different from meals in many other countries. The French typically eat their main meal mid-day [9] and the meal usually includes multiple courses (usually three), including cooked vegetables [10]. Similarly, French children usually have a hot lunch mid-day at home or at school, though mainly at school as part of institutional practices. School lunches are designed following dietary rules based on energy and balance of nutrients [11]. Lunches have multiple courses and always include vegetables as a either a starter and/or as a side dish. However, food waste is observed in school restaurants: the official French agency for the environment estimates food waste to be between 150 g and 200 g per person [12]. Detailed studies based on behavioral data are needed to better understand reasons for non-consumption and identify the potential levers to select optimum food offers.

### 1.1. Different Methods to Measure Waste

Most people probably view weighed food intake as the gold standard for measuring consumption – with whole meals or individual meal components being weighed before and after the meal. This method is time-consuming, labor-intensive, and requires space [13,14]. It is difficult to do weighed assessments with large samples of people or with complex meals with many components.

Alternatives to weighing food are visual estimation, i.e., direct observation by an experimenter, and photographic methods. Visual estimation has high reliability, requires less labor, less time, and less space [13,15], but requires trained observers [14]. Another method is digital photography where the estimation of amount consumed/amount wasted can be done at any time, and the photos can be checked for inter-judge reliability [3,16].

Byker Shanks, Banna, and Serrano (2017) [17] review 53 articles measuring nutrient intake in the United States National School Lunch Program. Different methods were used in the studies: visual estimation (*n* = 11), photography (*n* = 11), weighing (*n* = 23), or a combination of these three (*n* = 8). Most studies compared pre- and post-intervention in the school lunches. Fruits and vegetables were the most researched food category and the most wasted. The authors recommend more consistency in measuring food intake and food waste in school lunch assessments. For example, they note the wide variability in how food waste is reported, including grams, ounces, percentages, or kilocalories, with percentages being the most frequent. They also note the increasing popularity of visual estimation methods, including photography. Various scales have been used to visually estimate the food left in the plate as shown in Table 1.

### 1.2. Photographic Methods

Martin et al. (2014) [24] provide an overview of the photographic method of food consumption and waste estimation, noting its reliability and validity. Portion-size estimates using photography correlate highly with weighed food intake, although research shows that the estimated intake does vary over categories of nutrients but no information of potential link with food items is given. Additionally, the photographic method does not appear to change significantly across different raters, making it suited to large studies with multiple raters. The photographic method has been applied in a wide range of food service settings including different levels of schools (including preschool and elementary school).

The photography method has been used in various contexts. Christoph et al. (2017) [25] applied pre- and post-meal digital photography to measure dietary intake in a university self-serve dining-hall in the USA. They report high degrees of inter-rater agreement on selection, servings, and consumption of food groups, with the most challenge for consumption of mixed food items. Taylor et al. (2018) [26] extend photo analysis to student packed lunches in the USA, concluding that there was satisfactory reliability in assessment of quantities selected and consumed in eight food categories, with meat/meat alternatives providing the biggest challenge. Pouyet et al. (2015) [27] used a photographic method to study elderly patients’ meals in French nursing homes. A range of 11 reference images (0 to 100% food with 10% increments) was provided to judges to help evaluate food waste. They observed reliability of the method in that there was agreement between different untrained assessors and agreements among various estimates made by the same assessor. They confirmed the accuracy and specificity of this method by comparing food intake estimates for the four dishes with the food intakes determined using the traditional weighed food method. The method worked equally well on different food types. Hubbard et al. (2015) [28] used a photographic method to estimate waste and consumption in a nudge intervention study with intellectually and developmentally disabled children in a school setting.

Smith and Cunningham-Sabo (2013) [3] used a photographic method to study food waste in the US school lunch program. They estimated waste to the nearest 10% for the main dish, canned fruit, fresh fruit, vegetable, grain and milk. One-third to one-half of fruits and vegetables were wasted. Yoder, Foecke and Schoeller (2015) [4] studied waste following interventions in the US school lunch program, measuring trays pre and post meal. The percentage remaining on the tray was estimated in 25% increments (0%, 25%, 50%, 75% and 100%) of the served portion. Amounts wasted were calculated as the served portion multiplied by the percentage remaining. Results showed greater waste for raw fruits (compared to cooked), cooked vegetables (compared to raw), locally sourced (as compared to conventionally sourced, salad bar items (as compared to main menu items).

Byker Shanks et al. (2017) [2], as noted above, reported on 11 studies using digital photography in their assessment of the National School Lunch Program in the USA The studies used a variety of procedures, some studies using reference sizes of food components, and others using students’ selected food before consumption; in both cases, they reported percentage of the reference or the pre-consumption amount. Different studies used different percentage amounts (actual percent, increments of 10%, or 0%, 10%, 25%, 50%, 100%). Food waste was used to measure food waste and food consumption, as well as a number of interventions and procedures.

### 1.3. Food Consumption and Waste in School Canteens

Several studies researched different methods for studying the low selection and intake of vegetables in schools. Getts et al. (2015) [29] studied the quarter-waste method of visual estimation in a school cafeteria and reported good validity and reliability. Comparing the estimated weight to the actual weight, they found almost 90% of foods were in either “almost-perfect agreement (45%) or “substantial agreement” (42%). Comparing inter-rater reliability, they found over 90% in perfect to substantial agreement. Hanks et al. (2014) [13] also compared estimation methods (quarter-waste, half-waste, photograph) with weighed food leftovers. They noted problems with photograph estimation when foods are in containers, such as milk. The quarter-waste method reliability was slightly better than the half waste. The authors recommend the photograph method for estimation of waste of selected, unpackaged foods.

The goals of this research were to examine French children’s food waste in a school restaurant, to analyze consumption across the range of diverse foods composing a complete meal and explore reasons for non-consumption: the study analyzes actual waste in primary school and its link to taste preference. In addition to extending the use of photographic measurement of waste to the school setting in a natural environment, this research was designed to extend the study of school lunch to France which has a specific lunch pattern of eating (starter, main and side dish, dessert). To this end, a digital photography equipment was set-up in a typical French school to record leftovers at the tray disposal point. Children’s interviews were conducted on a subsample of participants to explore reasons of non-consumption.

## 2. Materials and Methods

### 2.1. General Procedure

The experiment took place in the natural setting of the school cafeteria. Observations were made at lunchtime. The school cafeteria was self service. On most days, children could choose between two cold-served vegetables served as a starter. Then they were served a main dish composed of meat/fish and a side, a dairy product (that they could choose), and a dessert (that they choose).

**Figure 1:**
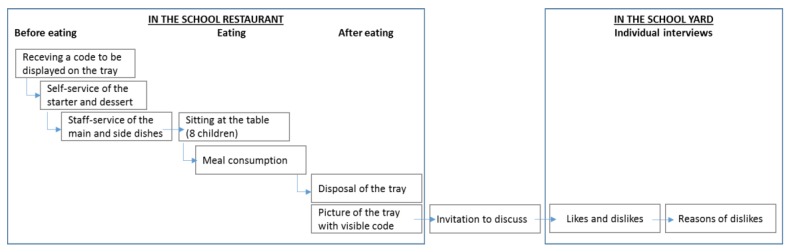
General scheme of the protocol with children.

The school was chosen in such a way that children were from a wide range of socio-economic status. Participants were children aged 6 to 12 who usually had lunch in the school canteen. All data were fully anonymous. Families pay for the meal according to their income. Tap water was the only available beverage. Menus are conceived in accordance with the national regulation GEMRCN ensuring dietary balance and hygiene standards. Food is prepared by a catering company in a central kitchen and delivered to the canteen using a cold chain (chilled food) scheme. The employees preparing and serving meals are city staff, trained to respect process and portion sizes. Children were given a code at the entrance of the school canteen to display on their tray. Then they behaved as usual until the disposal of their tray where a camera was set-up filming all trays during the service. At the entrance/exit of the school canteen, an experimenter was available to children to discuss the meal (Figure 1).

### 2.2. Ethics

The experiment received a favorable response from the ethics committee of the CHU of Lyon (Ref. Rech_FRCH_2013_005, Feb 6, 2013). Parents were informed about the study by the director of the school, who collected authorizations. None of the parents refused child participation in the study.

### 2.3. Participants

The data were collected through a 5-day longitudinal follow-up including a total of 215 children. Children of 4th and 5th grade classes were absent on one day because of a school trip. A total of 776 trays were analyzed with a good balance of gender (50.6% girls) and age as shown in Table 2.

### 2.4. Part 1 Waste

**Figure 2:**
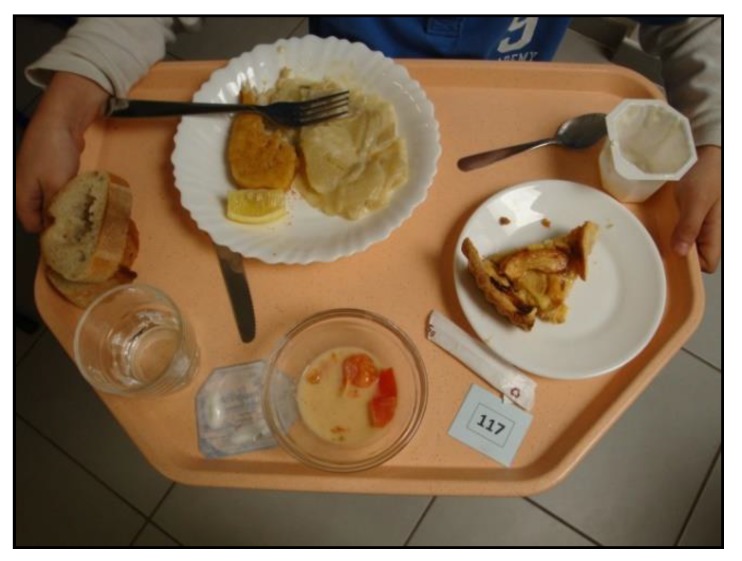
Example of the captured image of a disposed tray.

Two days of pre-tests were used to set up the cameras in the school canteen at the disposal area and to precisely define the process with the children: giving an individual label to be displayed on the tray and visible when disposing the tray after the meal. Digital images of disposed trays were automatically done with a camera set on a tripod with a distance of one meter at an angle of 45°. Pictures of foods were done after the meal (Figure 2). Extraction of a single image for each child was manually done remotely at the office.

For each child, each meal component was analyzed individually. The food remaining on the plate was evaluated as a percentage of the served quantity using a 5-point scale: 0, 1, 2, 3, or 4 corresponding to 0%, 25%, 50%, 75% or 100% of the served portion.

Waste was analyzed by food categories selected to be homogeneous in culinary terms: Raw vegetable (starter), Cooked vegetable served cold (starter), Starches (side dish), cooked vegetable served warm (side dish), main dish, dairy products, fruits, and desserts (Table 3).

### 2.5. Part 2 Liking

The second part of the methodology consisted of exploring children’s reactions to the served dishes.

A subgroup of approximately 50 children per day, 10 children per grade, was interviewed after meals to explore reasons for non-consumption and, more specifically, reasons for non-appreciation. A total of 248 children (62% girls) were randomly selected from the children who left food on their tray.

The interview was a semi-structured one with two sections, one for the starter and one for the main dish. The questions were: Which starter / main dish did you choose today? Did you enjoy it? Had you had enough? too much? not enough? If not liked, why didn’t you like it?

The interviews allowed us to focus on dislikes through the forced categorization into two liking categories (Liked or Disliked) and to develop reasons for dislikes.

A thematic analysis was conducted to interpret children discourses. Four categories were determined after a first reading of collected verbatim interviews. Three categories are sensory ones: appearance, taste and flavor, texture; one category concerns one food component often cited as rejection cause: the sauce.

### 2.6. Statistics

A one-way ANOVA was performed to evaluate the difference of waste scores and a Chi Square analysis to evaluate the proportion of likes and dislikes across the offered food range. A Pearson coefficient was calculated to describe the correlation between waste and liking based on the 7 analyzed food components (see Table 3). Statistical analyses were performed using XLSTAT 2015 (version 2015.4.01.22368, Addinsoft^TM^).

## 3. Results

### 3.1. Part 1. Waste

**Figure 3:**
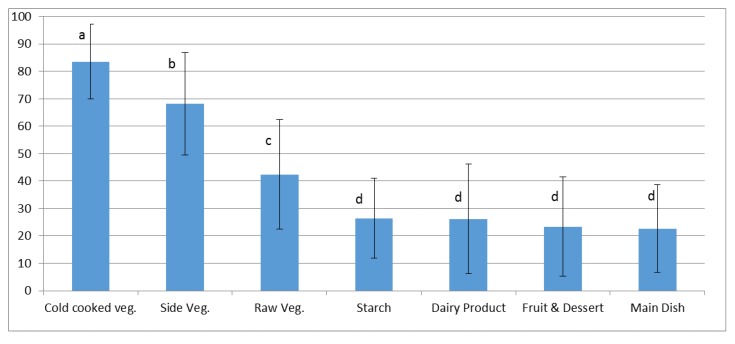
Mean rate of individual wastage of meal components (Error bars are standard deviations. Food with the same letter are not significantly different ANOVA 5% risk level).

Vegetable dishes were significantly the most wasted food (F(ddl = 6) = 132,812, *p* < 0.0001) especially cooked vegetables, either served warm as a side dish (66% wasted) or cold as a starter (83% wasted). Vegetable dishes were less wasted when the vegetable was raw (42% wasted), e.g., salad with dressing served as a starter. Other food components, main dish and starch or dairy product, fruit, and dessert, were rather well consumed with wastage rates between 22% and 25% (see Figure 3).

### 3.2. Part 2. Liking

**Figure 4:**
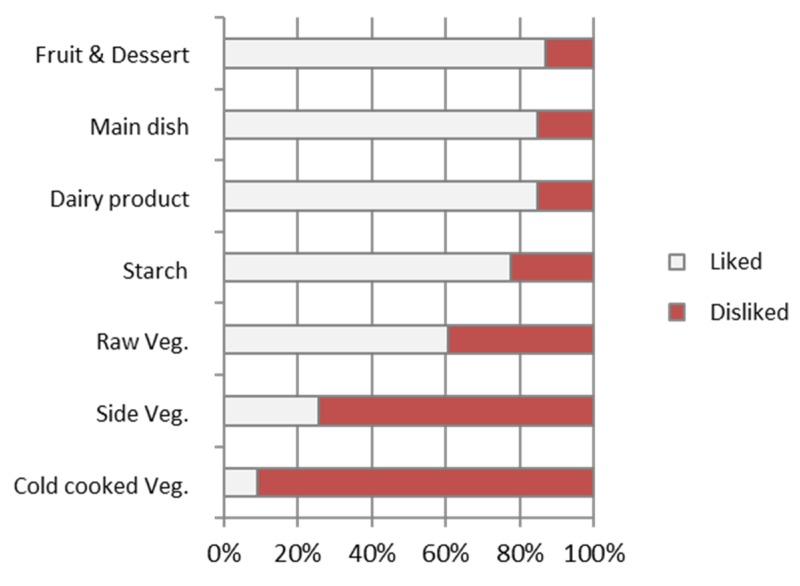
Categories of likes/disliked food components.

Interviewing children identified liked and disliked food components (Figure 4). The Chi Square statistics confirm observed differences (Chi²(6) = 317.16). Cooked vegetables served as starter or side dish were disliked whereas raw vegetables, main dishes, starches, dairy products, fruits and desserts were liked.

The thematic analysis of children’s discourse gave information on reasons for rejection. ‘Taste is the most-cited word, showing the importance of sensory characteristics in rejection. Two dishes were particularly disliked: the two recipes of cooked vegetable served as cold starters. For the leeks with dressing, taste mainly refers to texture of the slimy appearance. For the zucchini al pesto, it mainly refers to the unusual temperature (cold) and hardness. Moreover, both dishes are criticized regarding the sauce and the too-large quantity of sauce. Texture is often a major reason for food rejection [30].

Waste quantities are highly correlated with the liking status of foods. The Pearson coefficient of −0.997 (R^2^ = 0.994) is significant (*n* = 317, *p* < 0.0001).

## 4. Discussion

The present study was undertaken to study food selection and food waste in a typical French school lunch, with multiple courses of hot and cold foods, savory and sweet foods, using a photography method. Waste was measured using the 5-point quarter-waste scale [13]. As noted in previous research in the USA (see [2], for a review) and other countries [31], vegetables were the most wasted food category, especially cooked vegetables, which were wasted at rates of 66%–83%. Raw vegetables were still wasted more (42%) than main dishes, starch, dairy, fruit and desserts (22–25%).

Similarly, vegetables were the most disliked food category, with the following classes of vegetables in the same order as for waste: cold cooked vegetable > side cooked vegetable > raw vegetable. Reasons for liking/disliking included sensory dimensions (e.g., “slimy”) and other reasons. There was a high correlation between waste and liking (*p* =< 0.0001). There is a strong connection between food liking and food consumption, but there is not yet strong research evidence identifying food disliking as the major reason for waste. However, numerous authors point to the importance of personal preferences in generating food waste and hopefully consumption, can be improved with design of school menus more in line with student preferences.

This research supports the use of the 5-point quarter-waste method to measure food waste from photographs or online. Byker Shanks et al. (2017) [2] noted the problems arising from using a variety of scales to measure waste, and recommended more standardization of scales and methods.

This research also supports the photographic method for measuring food waste. As noted above, weighed assessments of each food portion before and after a meal is time-consuming and labor-intensive, while visual estimation of each food portion requires less time and labor, but requires trained observers. Both weighed assessments and visual estimation are difficult with large groups of people and with meals with many components. The photographic method is the only method where the meal images can be retained and checked later for validity and reliability. Further, the photographic method is not labor-intensive on the day of field testing; it is efficient in terms of space, personnel, and required training.

## 5. Conclusions

The future of waste measurement in schools and in other eating contexts will probably utilize automated analysis of food photographs. Future software will be able to automatically recognize food items and measure food proportions remaining from standard serving sizes. However, the challenge of measuring complex foods, complex food combinations, and foods in containers might continue. This will help foodservice teams to adjust the offer to liked and consumed food and lower food waste in the cafeteria. It also opens the path to build an educational program in school cafeterias to follow the effect of actions such as exposure to foods in conjunction with adjustable portion sizes of less consumed targeted food.

## Figures and Tables

**Table 1:** Diversity of scales used to measure food waste through visual estimation.

Scale	Authors	Food Left in the Plate
3-point scale	Kandiah et al. 2006 [18]	all; >50%; <50%
4-point scale	Hiesmayr et al. 2009 [19]	all; 1/2; 1/4; none
5-point scale	Graves et al. 1983 [20]	all; 3/4; 1/2; 1/4 or less; none or almost none
	Hanks et al. 2014 [13]	all; 3/4; 1/2; 1/4; none
6-point scale	Comstock et al. 1981 [21]	all; 1 bite eaten; 3/4; 1/2; 1/4; none
	Navarro et al. 2016 [22]	100%; 90%; 75%; 50%; 25%; 0%
7-point scale	Sherwin et al. 1998 [23]	all; 1 mouthful eaten; 3/4; 1/2; 1/4; 1 mouthful left; none
11-point scale	Williamson et al. 2003 [14]	100%; 90%; 80%; 70%; 60%; 50%; 40%; 30%; 20%; 10%; 0%

The 5-point scale—quarter-waste method – is recommended by Hanks et al. (2014) as the most reliable.

**Table 2:** Number of children by grade (boys/girls). * CLIS class (classe pour l’inclusion scolaire) is composed of children of different ages with school difficulties and training assistance.

	1st Grade	2nd Grade	3rd Grade	4th Grade	5th Grade	CLIS*	Total
Day 1	30 (14/16)	29 (13/16)	35 (20/15)	32 (13/19)	32 (18/14)	7 (4/3)	165
Day 2	30 (16/14)	37 (18/19)	29 (19/10)	17 (6/11)	35 (19/16)	0 (0/0)	148
Day 3	31 (12/19)	44 (19/25)	34 (20/14)	32 (14/18)	32 (18/14)	7 (5/2)	180
Day 4	35 (15/20)	47 (20/27)	32 (19/13)	35 (13/22)	28 (14/14)	8 (5/3)	185
Day 5	25 (11/14)	40 (16/24)	31 (21/10)	n/a	n/a	2 (1/1)	98
Total	151 (68/83) 19.4%	197 (86/111) 25.4%	161 (99/62) 20.7%	116 (46/70) 14.9%	127 (69/58) 16.4%	24 (15/9) 3.1%	776 (383/393) (50.6% girls)

**Table 3:** Food items and food categories served during the 5 observation days.

Food Categories	Food Items
1. Starter—Raw vegetable	Diced tomatoes Grated carrots Iceberg lettuce Radishes with butter Romaine Lettuce
2. Starter—Cooked vegetable served cold	Leeks Zucchini with pesto
3. Starches	Chickpea salad Creole Rice Pasta Salad
4. Side dish—Cooked vegetable served warm	Eggplant gratin Ratatouille Swiss Chard gratin
5. Main dish	Breaded fish (hoki) Fish filet (hoki) with cream sauce Fish filet (lieu noir) with sauce Plain Omelet Shell Pasta with 5-cheese tomato sauce Spaghetti Bolognaise
6. Dairy products	Blue cheese Cheese specialty Cottage cheese Emmental cheese Gouda Mixed Fruit Yogurt
7. Fruits and desserts	Apple Sauce Apple Tart Apricot Apricot Tart Fruit Cocktail Kiwi Orange Peach Vanilla Muffins Waffles

## References

[1] Appleton K., Hemingway A., Saulais L., Dinnella C., Monteleone E., Depezay L., Morizet D., Perez-Cueto F.J.A., Bevan A., Hartwell H. (2016). Increasing Vegetable Intakes: Rationale and Systematic Review of Published Interventions. Eur. J. Nutr..

[2] Byker Shanks C., Banna J., Serrano E.L. (2017). Food Waste in the National School Lunch Program 1978–2015: A Systematic Review. J. Acad. Nutr. Diet..

[3] Smith S.L., Cunningham-Sabo L. (2013). Food choice, plate waste and nutrient intake of elementary- and middle-school students participating in the US National School Lunch Program. Public Health Nutr..

[4] Yoder A.B.B., Foecke L.L., Schoeller D.A. (2015). Factors affecting fruit and vegetable school lunch waste in Wisconsin elementary schools participating in Farm to School Programmes. Public Health Nutr..

[5] Moreno-Black G., & Stockard J. (2018). Salad bar selection patterns of elementary school children. Appetite.

[6] McIntosh W.A., Dean W., Torres C.C., Anding J., Kubena K.S., Nayga R., Meiselman H.L. (2009). The American Family Meal. Meals in Science and Practice.

[7] Makela J., Kjaernes U., Ekstrom P., Kjaernes U. (2001). What did they eat. Eating Patterns: A Day in the Lives of the Nordic Peoples.

[8] Makela J., Kjaernes U. (2001). The meal format. Eating Patterns: A Day in the Lives of the Nordic Peoples.

[9] de Saint Pol T. (2005). Quand est-ce qu’on mange? Le temps des repas en France. Terrains et Travaux.

[10] Fischler C., Masson E. (2008). Manger: Français, Européens et Américains face à L’alimentation.

[11] Ministère de l’économie, de l’Industrie et du numérique (2015). Recommandation Nutrition. Groupe d’Etude Des Marchés de Restauration Collective et Nutrition GEM-RCN.

[12] ADEME Réduire le gaspillage alimentaire en restauration collective (2018). Guide Pratique de L’agence de et la Maîtrise de L’énergie.

[13] Hanks A.S., Wansink B., Just D.R. (2014). Reliability and Accuracy of Real-Time Visualization Techniques for Measuring School Cafeteria Tray Waste: Validating the Quarter-Waste Method. J. Acad. Nutr. Diet..

[14] Williamson D.A., Allen H.R., Martin P.D., Alfonso A.J., Gerald B., Hunt A. (2003). Comparison of digital photography to weighed and visual estimation of portion sizes. J. Am. Diet. Assoc..

[15] Martins L.M., Cunha L.M., Rodrigues S.P., Rocha A. (2014). Determination of plate waste in primary school lunches by weighing and visual estimation methods: A validation study. Waste Manag..

[16] Sabinsky M.S., Toft U., Andersen K.K., Tetens I. (2013). Validation of a digital photographic method for assessment of dietary quality of school lunch sandwiches brought from home. Food Nutr. Res..

[17] Byker C.J., Farris A.R., Marcenelle M., Davis G.C., Serrano E.L. (2014). Serrano Food Waste in a School Nutrition Program After Implementation of New Lunch Program Guidelines. J. Nutr. Educ. Behav..

[18] Kandiah J., Stinnett L., Lutton D. (2006). Visual plate waste in hospitalized patients: Length of stay and diet order. J. Am. Diet. Assoc..

[19] Hiesmayr M., Schindler K., Pernicka E., Schuh C., Schoeniger-Hekeler A., Bauer P., Laviano A., Lovell A., Mouhieddine M., Schuetz T. (2009). Decreased food intake is a risk factor for mortality in hospitalised patients: The Nutrition Day survey 2006. Clin. Nutr..

[20] Graves K., Shannon B. (1983). Using visual plate waste measurement to assess school lunch food behaviour. J. Am. Diet. Assoc..

[21] Comstock E.M., St Pierre R.G., Mackiernan Y.D. (1981). Measuring individual plate waste in school lunches. Visual estimation and children’s ratings vs. actual weighing of plate waste. J. Am. Diet. Assoc..

[22] Navarro D.A., Boaz M., Krause I., Eli A., Chernov K., Giabra M., Frishman S., Levy M., Giboreau A., Kosak S. (2016). Improved meal presentation increases food intake and decreases readmission rate in hospitalized patients. Clin. Nutr..

[23] Sherwin A., Nowson C., McPhee J., Alexander J., Wark J., Flicke L. (1998). Nutrient intake at meals in residential care facilities for the aged: Validated visual estimation of plate waste. Aust. J. Nutr. Diet..

[24] Martin C.K., Nicklas T., Gunturk B., Correa J.B., Allen H.R., Champagne C. (2014). Measuring food intake with digital photography. J. Hum. Nutr. Diet..

[25] Christoph M.J., Loman B.R., Ellison B. (2017). Developing a digital photography-based method for dietary analysis in self-serve dining settings. Appetite.

[26] Taylor J.C., Sutter C., Ontai L.L., Nishina A., Zidenberg-Cherr S. (2018). Feasibility and reliability of digital imaging for estimating food selection and consumption from students’ packed lunches. Appetite.

[27] Pouyet V., Cuvelier G., Benattar L., Giboreau A. (2015). A photographic method to measure food item intake. Validation in geriatric institutions. Appetite.

[28] Hubbard K.L., Bandini L.G., Folta S.C., Wansink B., Eliasziw M., Must A. (2015). Impact of a Smarter Lunch- room intervention on food selection and consumption among adolescents and young adults with intellectual and developmental disabilities in a residential school setting. Public Health Nutr..

[29] Getts K.M. (2015). Measuring Plate Waste: Validity and Inter-Rater Reliability of the Quarter-Waste Method. Master’s Thesis.

[30] Egolf A., Siegrist M., Hartmann C. (2018). How people’s food disgust sensitivity shapes their eating and food behaviour. Appetite.

[31] Falasconi L., Vittuari M., Politano A., Segre A. (2015). Food Waste in School Catering: An Italian Case Study. Sustainability.

